# Characterization of the age-dependent intervertebral disc changes in rabbit by correlation between MRI, histology and gene expression

**DOI:** 10.1186/1471-2474-12-147

**Published:** 2011-07-04

**Authors:** Johann Clouet, Marianne Pot-Vaucel, Gaël Grimandi, Martial Masson, Julie Lesoeur, Borhane H Fellah, Olivier Gauthier, Marion Fusellier, Yan Cherel, Yves Maugars, Jérôme Guicheux, Claire Vinatier

**Affiliations:** 1 INSERM (Institut National de la Santé et de la Recherche Médicale) U791, LIOAD, Group Skeletal Tissue Engineering and Physiopathology (STEP), University of Nantes, Nantes F-44042, France; 2 PresUNAM, University of Nantes, Nantes, France; 3 Department of Pharmacy, University Hospital of Nantes, Nantes F-44093, France; 4 Department of Rheumatology, University Hospital of Nantes, Nantes F-44093, France; 5 Department of experimental Surgery, CRIP-VC, National Veterinary School (ONIRIS), Nantes F-44307, France; 6 Department of Imagery, CRIP-VC, National Veterinary School (ONIRIS), Nantes F-44307, France; 7 INRA UMR703, National Veterinary School (ONIRIS), Nantes F-44307, France; 8 Graftys SAS, Aix en Provence, France

## Abstract

**Background:**

The present study was conducted to address whether the intervertebral disc of rabbit could be considered (i) as a valuable model to provide new insights into the tissue and cellular changes of Nucleus pulposus aging and (ii) as an appropriate tool to investigate the efficacy of Nucleus pulposus cell-based biotherapies.

**Methods:**

Lumbar intervertebral disc from rabbits with increasing ages (1, 6 and 30 month-old) were compared by MRI and histological observation using Pfirrmann's grading and Boos' scoring respectively. The expression of transcripts (COL2A1, AGC1, COL1A1, MMP13, BMP2, MGP and p21) in Nucleus pulposus cells were analysed by quantitative real-time PCR.

**Results:**

MRI analysis indicated an early age-dependent increase in the Pfirrmann's grading. Histological Boos' scoring was also increased. The analysis of transcript expression levels showed that COL2A1 and AGC1 were down-regulated as a function of age. Conversely, COL1A1, MMP-13, BMP-2, MGP and p21 were significantly up-regulated in the Nucleus pulposus cells of aged rabbit intervertebral disc.

**Conclusions:**

Our study describes the consistency of the rabbit as a model of intervertebral disc changes as a function of age by correlating tissue alteration with cellular modification measured.

## Background

The intervertebral disc (IVD) lies between the vertebral bodies and links them together. The components of the disc are Nucleus pulposus (NP), Annulus fibrosus (AF) and the end-plates. Although the phenotype of IVD cells and the composition of the extracellular matrix (ECM) is still the subject of considerable debate, they appear quite comparable to those of articular cartilage particularly for NP cells [1,2]. This composite nature of IVD endows the disc with both the tension-resisting properties of a ligament and the compression-resisting properties of articular cartilage. Unfortunately, disc structure and function does not remain optimal throughout life, but undergoes a progressive age-dependent degeneration. The IVD aging initiates early in the NP, as seen by a loss of cellularity and alteration of the ECM, thus compromising the mechanical properties of IVD [3]. It is well acknowledged that IVD degeneration encompasses several age-related processes influenced primarily by mechanical, nutritional and genetic factors. However, the underlying cellular and molecular mechanisms involved in the premature initiation and progression of IVD aging and degeneration still remains poorly deciphered (for review see [4]). In this context, the development of appropriate animal models capable of providing new insights into the IVD physiopathology should be further investigated.

IVD blood supply terminates at the end-plate, making NP and AF non-vascularized tissue [3]. In addition, IVD is a poorly cellularized tissue [3]. Both these characteristics are responsible for the limited intrinsic repair capacity of IVD. Accordingly, IVD damages are irreversible and often result in clinical symptoms, such as low back pain, that require medical intervention [5]. Such treatments currently involve removal or replacement of the injured disc by surgery rather than its repair, which would be the preferred course of action. Successful transplantation of IVD autografts, allografts (fresh and fresh-frozen) have also been considered in primate models and in humans [6]. However the safety and efficiency of such techniques remain to be clarified. In this context, the use of cell-seeded biomaterials for tissue engineering of the IVD has been recently investigated [7,8]. Although results thus far are promising, the development of an in vivo model that can closely mimic human IVD aging and degeneration is crucial to test the efficacy of such future regenerative cell-based biotherapies. Among the different animal models described in the literature [9,10], the use of the rabbit disc model because in addition to being cost effective and the most widely investigated, it also appears to be a relevant model of age-linked altered proteoglycan metabolism and disc injury.

The present study was conducted to address whether the rabbit could be considered (i) as a valuable model to provide new insights into the tissue and cellular changes occurring during IVD aging and degeneration and (ii) as an appropriate tool to investigate the efficacy of IVD biotherapies. To this end, lumbar IVD from rabbits with increasing ages (1, 6 and 30 months old) were compared by MRI and histological staining. In an effort to determine whether a close correlation may exist between the tissue and cellular changes observed during the early course of aging, we also analyze the variation of transcript expression in NP cells with increasing age.

## Methods

### Animals and surgical procedures

All animal handling and surgical procedures were conducted according to European Community guidelines for the care and use of laboratory animals (DE 86/609/CEE). New Zealand White rabbits (Charles River, L'Arbresle, France), 1, 6 and 30-month old, were used. The study protocol has been approved by the ethics committee at the National Veterinary School of Nantes (ENVN-ONIRIS). Three rabbits per age group were used to perform MRI and histological analyses as well as five additional rabbits per age group for the RT-PCR analysis.

### MRI scanning and image assessment

MRI scans were performed using a one Tesla clinical magnet (Siemens Magnetom Harmony/Syngo). One, six, 30 month old rabbits (n = 3 for every ages) were anesthetized by intramuscular injection of xylazine (Bayer, Puteaux, France) and ketamine (Merial, Lyon, France). A 2.5 mm midsagittal section image was obtained, using a T2-weighted imaging sequence (T2ws) (TR, 5000 milliseconds; TE, 111 milliseconds).

Images were analysed by Pfirrmann's grading [11]. This grading system is based on MRI signal intensity, disc structure, distinction between NP and AF and disc height on T2-weighted magnetic resonance images. The degree of disc degeneration was ranked from grade 1 (normal disc) to grade 5 (severe degeneration) as previously described [11]. An evaluation of MRI scans was performed by three independent investigators expert in MRI image reading.

### Histological analysis

After rabbit euthanasia (n = 3 for every age group), the lumbar IVD were collected from five consecutive levels (L2-3, L3-4, L4-5, L5-6 and L6-7). IVD were dissected [2] and fixed in 10% paraformaldehyde for four days, decalcified for 24 hours in Decalcifier II^® ^(Surgipath, Richmond, USA). After dehydration and incubation with Histosol^® ^(Shandom, Brussels, Belgium), specimens were embedded in paraffin and sectioned into 3-μm slices. For histological analysis, 3 μm thick paraffin sections were deparaffined using toluene, rehydrated through a graded series of ethanol, and rinsed in distilled water. Sections were stained with hematoxylin phloxin safran (HPS) and with 0.1% alcian blue (Sigma-Aldrich, St Louis, USA) [12].

Histological sections were analyzed using Boos' modified scoring [13]. Four parameters were specifically assessed to classify age-related changes in IVD: cell density, mucous degeneration, tears and cleft formations and granular changes [13]. Each parameter was ranked from 0 to 4 according to the intensity of the tested parameters (0: lowest; 4: highest). A blind evaluation of histological samples was performed by three independent investigators expert in reading histological slides.

### Transcript expression analysis

Five rabbits of each age were sacrificed. NP tissues from five consecutive lumbar levels (L2-3, L3-4, L4-5, L5-6 and L6-7) were isolated and enzymatically digested as previously described [2]. NP cells were frozen for subsequent real time PCR analysis. Total RNA was extracted using Trizol^® ^reagent according to the manufacturer's instructions. Real-time PCR experiments were conducted as described extensively in [2]. Briefly, after deoxyribonuclease I digestion, RNA samples (2 μg) were reverse-transcribed using AMV-RT and random primers in a total volume of 30 μl. Complementary DNA (cDNA) was amplified in a total volume of 25 μl PCR reaction containing 12.5 μl of brilliant SYBR Green Master Mix (1X) and 30 nM of SYBR Green reference dye. The sequence of each primer (MWG Biotech, Ebersberg, Germany) set used are given in Table 1. Real-time PCR was performed using the Mx3000P QPCR system (Stratagene, La Jolla, CA, USA). Cycle thresholds were normalized to β-actin in order to correct for potential cDNA quantification differences. The expression levels of each gene were normalized to the average of all the 1 month-old rabbits and the results are reported as fold change in gene expression.

**Table 1:** real-time PCR investigation.

Gene (Abbreviation)	Accession number (Gene Bank)	Forward primer (5'-3')	Reverse primer (5'-3')	Product length
*GAPDH*	NM_002046	agccacatcgctcagaca	gcccaatacgaccaaatcc	66 pb

Type II collagen *COL2A1*	D83228	acagcaggttcacctataccg	cccacttaccggtgtgtttc	60 pb

Aggrecan *AGC1*	L38480	gaggatggcttccaccagt	tggggtacctgacagtctga	61 pb

Type I colagen *COL1A1*	D49399	agcgatggtcctccaggt	gccagggtaaccacgttct	63 pb

Matrix metalloproteinase 13 *MMP13*	NM_001082037	ttttgaagacacgggcaag	tcatcatagctccagacttggtt	60 pb

Bone Morphogenic Protein 2 *BMP2*	NM_001082650	tgcagaacttcaggtttttcg	tggaaaccgctgtcgtct	63 pb

Matrix Gla protein *MGP*	D21265	tggatataatgctgcttacaatcg	tttccaatcttattcagctctgc	64 pb

P21-activated protein kinase I *P21*	NM_001082756	agaaagaaaaagaacgaccagaga	cgtggatggtgtgctcaa	60 pb

GenBank reference of the genes evaluated and oligonucleotide primers used for real-time PCR.

### Statistical analysis

Each analysis was performed on three rabbits per age group for MRI and histology and on five rabbits per age group for RT-PCR. Results are expressed as mean +/- SD of triplicate samples. Comparative studies of means were performed using one-way ANOVA followed by a post hoc test (Fisher's projected least significant difference) with a statistical significance at p < 0.05.

## Results

### MRI and histological characterization of IVD aging

**Figure 1:**
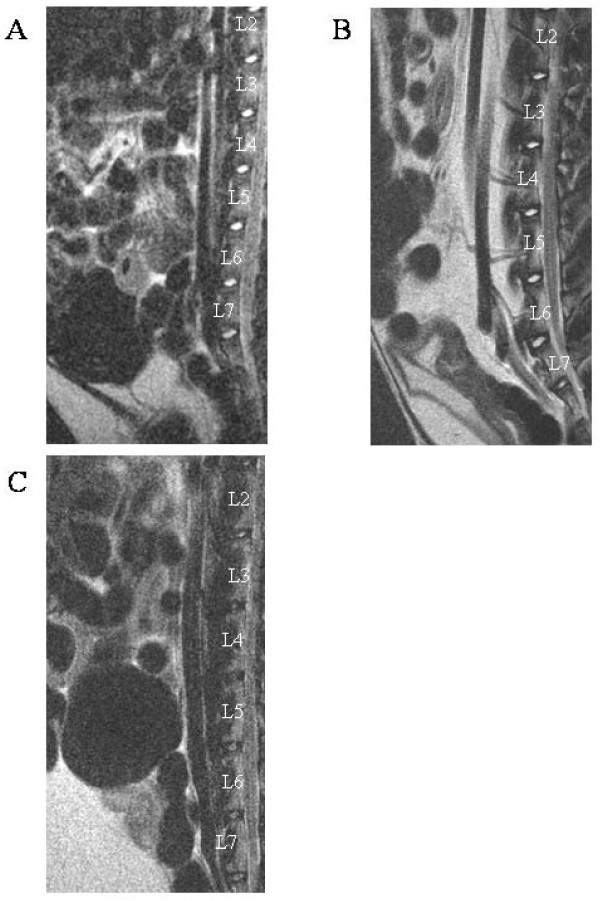
**MRI images of the lumbar spine of rabbits with increasing ages**. T2-weighted midsagittal images of (A) one-, (B) six- and (C) 30-month-old rabbits. Representative MRI images are shown.

In order to characterize the aging process of IVD, the T2ws intensity of lumbar spine from one, six and 30-month-old rabbits was assessed. Representative MRI picture of one-month-old IVD on Figure 1A indicates a grade I according to the Pfirrmann'grading to IVD from L2 to L6 levels. The lowest lumbar IVD (L6-L7) appears non-homogeneous with a dark transverse band. A grade II was assigned to this disc. The six-month-old rabbits' (Figure 1B) NP from L2 to L7 level showed a nonhomogeneous T2ws intensity with the presence of a dark transverse band and were assigned as grade II. Six-month-old disc height was not altered as compared to that of one-month old disc but the distinction between NP and AF became less discernable. For the 30-month-old rabbits (Figure 1C), all the IVD at lumbar level exhibited a non-homogeneous T2 signal intensity (gray). The IVD height was slightly decreased, and no distinction between NP and AF could be observed. These were scored as grade III. In summary, MRI data indicate that rabbits exhibit scalable IVD changes evolving as early as one-month old.

**Figure 2:**
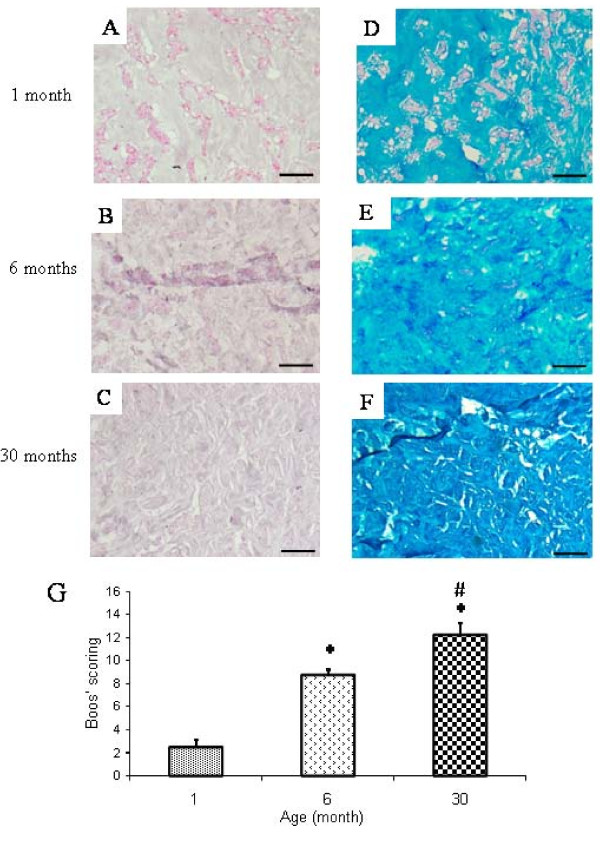
**Histological characterization of the *Nucleus pulposus *from IVD of rabbits with increasing ages**. Histological sections of IVD from (A,D) one-, (B,E) six-, (C,F) 30-month-old rabbits were stained with (A, B, C) Haematoxilin Phloxin Safran and (D, E, F) alcian blue. Bar: 50 μm. Representative images are shown. (G) A Modified Boos's scoring was used to assess quantitatively the age-dependent tissue changes. *: p < 0.05 as compared to 1-month old rabbits; #: p < 0.05 as compared to 6-month old rabbits.

To further address whether the age-dependent MRI changes of rabbit IVD may correlate with some tissue alteration, we then performed histological stainings. Analysis of histological staining revealed a dramatic decrease in cell density as a function of age (Figure 2A, B, C). In addition, mucous degeneration and granular changes, shown by the presence of large decoloured and heterogeneous areas in granulated regions, were markedly increased (Figure 2D, E, F). Finally, the formation of tears and clefts shown by the presence of small thin defects was also affected by aging. To quantitatively assess the histological damage observed as a function of age, we next used a modified Boos' scoring [13] (Figure 2G). Our results show a significant increase in the modified Boos' scoring from one to 30 months old. This scoring was 5.5-times higher in the 30-month-old rabbits compared to one-month old rabbits (p < 0.05).

### Alteration of transcript expression patterns during IVD aging

**Figure 3:**
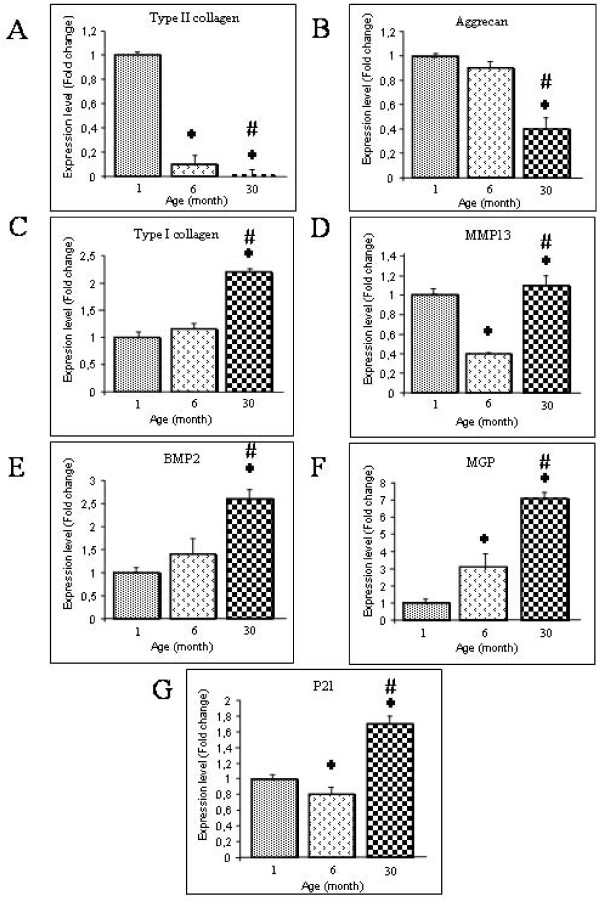
**Phenotypic markers in *Nucleus pulposus *cells**. Analysis of the expression levels of transcripts coding for the phenotypic markers (A) type II collagen, (B) Aggrecan, (C) type I collagen, (D) MMP-13, (E) BMP-2, (F) MGP and (G) p21 in Nucleus pulposus (NP) cells from IVD of rabbits with increasing ages. Messenger RNAs were purified from freshly isolated NP cells and analyzed by real-time PCR as described in the materials and methods section. Results are reported as fold change in gene expression related to one month old rabbits. *: p < 0.05 as compared to one-month-old rabbits; #: p < 0.05 as compared to six-month old rabbits.

To address whether the structural age-related changes identified by MRI and histology may be related to alteration of cell phenotype, we analysed the expression levels of transcript coding for several IVD associated genes in freshly isolated NP cells from increasing ages. We first focused our attention on transcript coding for ECM genes (type II collagen COL2A1, type I collagen COL1A1 and aggrecan AGC1). Our data indicated that COL2A1 mRNA levels decreased significantly with aging in NP cells (Figure 3A). The COL2A1 mRNA level was 100-fold lower in the 30-month-old rabbit compared to that of a one-month-old rabbit (p < 0.05). In parallel, a significant 2.5-fold decrease in AGC1 mRNA levels was also noted between one and 30-month-old rabbits (p < 0.05) (Figure 3B). In contrast to the decrease in COL2A1 and AGC1 mRNA levels, the COL1A1 mRNA level increased significantly with aging in NP cells (Figure 3C) with a 2.5-fold increase in COL1A1 mRNA at the age of 30-months compared to one-month. Taken together, these data indicate that the expression of chondrogenic markers (COL2A1 and AGC1) decreases with age whereas that of the dedifferentiation marker (COL1A1) increases as early as thirty months.

Since it has been reported that the expression of matrix metalloprotease 13 (MMP13), bone morphogenetic protein-2 (BMP2) and the matrix Gla protein (MGP) were affected during the course of osteoarthritis [14] and IVD aging [15], we were interested in deciphering whether these genes may also be affected by aging in rabbit NP. Whilst a significant decrease in MMP-13 mRNA levels was found between one and six-month-old NP, our data indicate that MMP13 mRNA levels increased significantly between 6 and 30-month old rabbit (2.8-fold higher) (p < 0.05) (Figure 3D). In addition, BMP2 mRNA levels was found to be significantly up-regulated between one and 30-month-old rabbits with a 2.5 fold increase (p < 0.05) (Figure 3E). MGP mRNA levels were significantly increased as a function of age with a significant seven-fold increase between one-and 30-month-old rabbits (Figure 3F).

Finally, since IVD aging has recently been associated with cellular senescence [16], we investigated whether the level of p21 mRNA may vary as a function of age (Figure 3G). Interestingly, our transcript analysis revealed that p21 mRNA level was significantly up-regulated (2 fold increase) between six month and 30-month-old rabbits.

Viewed together, our data strongly suggest that the age-dependent tissue changes evidenced by MRI and histology were associated with alteration of the phenotype of NP cells.

## Discussion

The current therapeutic strategies for patients with lower back pain remain symptomatic and are mainly dedicated to relieving painful symptoms. Our current understanding of the physiopathology of IVD degeneration allows us to consider regenerative medicine as a promising strategy. Indeed, recent reviews have considered the imbalance between anabolism and catabolism as a pivotal factor of the IVD degeneration process. This imbalance between anabolism and catabolism contributes to the disorganization of the ECM. Accordingly, increasing attention has been paid to the regeneration of functional tissue based on the restoration of the ECM integrity by cell therapy and/or tissue engineering [3,17]. Nevertheless, before these promising biotherapies may enter the therapeutic arsenal, some preclinical tests in adapted animal models that closely mimic the physiological aging and degeneration process of human IVD should be performed. In this context, the present study aims at characterizing IVD aging and degeneration in the rabbit to propose this model as a suitable tool to i) gain new insights in the complex mechanisms of IVD degeneration and ii) to perform preclinical experiments dedicated to the evaluation of the safety and efficacy of IVD tissue engineering strategies.

In humans, MRI is the gold standard for the clinical investigation of IVD integrity. This technique allows the definition of IVD based on the tissue hydration shown by the intensity of the T2ws in the NP. The pictures of rabbit lumbar spines showed an age-dependent decrease in the T2ws and the appearance of a dark transverse band. This data is quite similar to that observed in humans during the course of IVD degeneration [18]. The decrease in T2ws intensity in human IVD after 20 years of age is well-acknowledged and reflects the decrease in water and proteoglycan contents during aging [19]. The dark transverse band is considered to be a normal structure in persons of 30 years or older [18]. Histological studies have thus suggested that this dark transverse band may likely to be due to the accumulation of fibrous tissue in the central zone of NP [20]. To precisely assess disc degeneration on routine T2ws in human, Pfirrmann et al. have developed a reliable grading system [11]. Interestingly, the use of Pfirrmann's system to grade IVD degeneration in rabbit reveals the same profile of T2ws variation as a function of age, thereby confirming the reliability of the different periods of life in rabbits previously described by Murakami et al. [21]: grade I for childhood (one-month old in rabbits is similar to a child in humans), grade II for adolescent rabbit (six months old in rabbits is similar to an adolescent in humans) and grade III-IV in adult age (30 months old in rabbits is similar to an adult in humans). All these MRI data suggest that the age-related changes that occurs in rabbit IVD share consistent similarities with those traditionally observed during the course of human IVD aging.

To further address whether the age-related changes observed by MRI may correlate with some tissue alteration, we then performed histological stainings. We were interested in addressing whether the modified Boos' scoring, originally developed to histologically assess IVD changes in humans, could be transposed to rabbit IVD aging. Accordingly, the main features of human IVD degeneration were found in rabbit IVD with mucous degeneration, granular changes as well as cracks and tears being formed as a function of age. Interestingly, these histological changes perfectly corroborate the changes observed by MRI (loss of T2ws intensity and formation of a dark band). Taken together, our MRI and histological data show some similarities between the process of IVD aging in rabbits and humans thereby strongly suggesting that rabbits could be considered as a valuable spontaneous model of age-dependent IVD changes. In addition to being a reliable model of IVD aging, this spontaneous model may provide a closer representation of the pathophysiology of IVD aging in humans compared to the degeneration induced by needle aspiration or chemonucleolysis [9]. Despite our data strongly suggesting that the rabbit is a relevant model of IVD aging, some limitations should, however, be considered. Contrarily to humans, rabbits have notochordal cells in their NP at least up to 6 months of age [22]. In addition, in small quadrupeds and despite a relative discrepancy in disc diameter, the spine is likely to be loaded with smaller forces as compared to humans [23,24]. Whether both of these limits affect the relevance of the rabbit model is not yet known.

To our knowledge, only two studies have partially described IVD aging in rabbits [25,26]. In these previous studies, rabbit IVD aging was evaluated longitudinally between the age of 6 and 42 months. In this previous study, rabbit IVD aging is longitudinally evaluated between the age of six and 42 months. On the contrary to our study, no result is available for the period ranging from one to six months, which could be an essential period for the onset of IVD aging. In fact, it is well known that the NP of less than twelve-month-old rabbits contain some remnant cells originating from the embryonic notochord [22]. Whether the age-dependent loss of notochordal cells, most likely by apoptosis, is intimately related to IVD aging is not yet fully deciphered but has been proposed as a possible initiating mechanism for IVD degeneration [27].

NP is the structure in which IVD degeneration is initiated by dehydration followed by an alteration of the structural organization of the tissue [13]. Therefore, to strengthen our MRI and histological data, we finally sought to decipher whether the above described age-dependent tissue changes may correlate with alteration in the NP cell phenotype. To address this issue, we focused our attention on the expression levels of molecules that were previously shown to be modulated during the onset of IVD degeneration or osteoarthritis (COL1A1, COL2A1, AGC1, BMP2, MMP13, MGP and P21) [14,16,28]. Since it remains difficult to quantitatively evaluate the level of the corresponding proteins, particularly in the rabbit, we embarked on the analysis of the corresponding transcript levels by real-time RT-PCR. Our data describing an age-dependent increase in COL1A1 expression with a concomitant decrease in COL2A1 and AGC1 expression suggest that NP cells experience a process of dedifferentiation. Interestingly, this dedifferenciation process has been well described in cultured articular chondrocytes [29] and in osteoarthritic joints [30]. Our data also suggests that during the process of IVD aging, the molecules with a longer half-life (collagen type II) exhibit an early decrease in the corresponding transcript levels. Conversely, the molecules with a shorter half-life (aggrecan) have a transcript expression levels that starts to decline later on. Whether this differential regulation of ECM molecules may be of importance for the maintenance of IVD integrity deserves further attention.

Among the various genes modulated in osteoarthritic chondrocytes [14], MMP-13 is probably the most trustworthy. We were therefore interested to find a significant modulation of these genes during IVD degeneration. MMP-13 is known to degrade collagens and glycosaminoglycans [31]. The increase in MMP13 could therefore be a major contributor of IVD degeneration [16] as it has been extensively reported in cartilage degradation during OA [14].

BMP-2, a growth factor that stimulates the production of ECM components in IVD [21] and articular cartilage [32], was also shown to be stimulated as a function of age in rabbit IVD. One can assume that the increase in BMP2 expression during IVD aging is related to the existence of reparative mechanisms that could contribute to slowing down ECM degradation. In line with this suggestion, MGP, one of the most potent inhibitors of calcification in mammals [33], was also found to be up-regulated as a function of age [15] thereby supporting our hypothesis of the existence of such compensatory mechanisms. Of note, similar compensatory mechanisms have been described in osteoarthritic cartilage, which draws attention to the physiopathological vicinity of IVD degeneration and osteoarthritis. Finally, the age-dependent increase in p21, a cycline dependant kinase inhibitor that in addition to being involved in cellular senescence [16] has also been reported to inhibit type II collagen expression in articular chondrocytes [34], further highlights the similarities between OA and IVD degeneration.

## Conclusions

This study describes the consistency of the rabbit as a model of age-dependent IVD changes. Our data also highlight some similarities between the physiopathological processes involved in the onset of IVD aging and those described for osteoarthritis. Finally, our study makes the rabbit a valuable tool (i) to gain new insights into the complex molecular mechanisms that govern IVD aging and (ii) to test the preclinical efficacy of tissue engineering strategy that may offer the possibility of regenerating damaged IVD.

## Competing interests

The authors declare that they have no competing interests.

## Authors' contributions

JC and MPV have conceived and coordinated the study. They participated in MRI analysis, Boos'scoring and RT-PCR analysis. They also performed the statistical analysis and drafted the manuscript. MM performed the PCR analysis. JL performed the histological stainings. BHF, OG participated in the design of the study, particularly in animal management. MF performed MRI imaging and participated in analysis. YC participated in histological analysis. GG and YM have helped in the writing of the manuscript. JG and CV helped to the conception of the study, and participated in its design and have helped in the writing of the manuscript. All authors read and approved the final manuscript.

## Pre-publication history

The pre-publication history for this paper can be accessed here:

http://www.biomedcentral.com/1471-2474/12/147/prepub
